# Adoption of Improved Reprocessing Decreased Microbiological Non-Compliance for Bronchoscopes

**DOI:** 10.3390/ijerph192113978

**Published:** 2022-10-27

**Authors:** Benedetta Tuvo, Michela Scarpaci, Tommaso Cosci, Alessandro Ribechini, Silvia Briani, Grazia Luchini, Michele Totaro, Angelo Baggiani, Maria Luisa Cristina, Simona Barnini, Simone Leonetti, Beatrice Casini

**Affiliations:** 1 Department of Translational Research and the New Technologies in Medicine and Surgery, University of Pisa, 56126 Pisa, Italy; 2 Thoracic Endoscopy Division, University Hospital of Pisa, 56126 Pisa, Italy; 3 Hospital Management, University Hospital of Pisa, 56126 Pisa, Italy; 4 Department of Health Sciences, University of Genova, Via Pastore 1, 16132 Genova, Italy; 5 Microbiology Unit, University Hospital of Pisa, 56126 Pisa, Italy; 6 Department of Life Science, School of Advanced Studies Sant’Anna, 56124 Pisa, Italy

**Keywords:** endoscope reprocessing, bronchoscopes, microbiological surveillance, NDM-producing *Klebsiella pneumoniae*

## Abstract

Background: In the past few decades, the inadequate reprocessing of bronchoscopes has been associated with several serious outbreaks caused by multidrug-resistant microorganisms. In this study we evaluated the improvement in the quality of reprocessing in a Bronchoscopy Unit (BU), after the introduction of a new procedure. Methods: In 2019, observational and clinical audits were conducted in the BU. After the introduction of an improved procedure in 2020, a microbiological surveillance plan was implemented in 2021. Results: In 2019, 13 of 22 bronchoscopes (59%) resulted as non-compliant, 18% as high concern organisms (HCO) and 36.4% as high microbial count (≥100 CFU/all channels) and HCO. The most frequent microorganisms were *Staphylococcus aureus* (38.5%) and NDM-producing *Klebsiella pneumoniae* (15.4%). The bronchoscopes were stored inside their transport cases, which in some cases were found to be contaminated by the same strains isolated on the bronchoscopes (*Enterobacter gergoviae* and *Vibrio alginolyticus*). In 2021, all 31 bronchoscopes were sampled at least three times and 13/99 (13.1%) resulted as non-compliant, mostly *K. pneumoniae* (4.04%). Contamination level increases weakly in bronchoscopes in use for more than 14 years (R = 0.32). Conclusions: The adoption of an improved reprocessing procedure decreased the non-compliance of bronchoscopes, increasing the quality of the process and patient safety.

## 1. Introduction

Bronchoscopy is a diagnostic and therapeutic procedure widely used for different diseases of nasopharynx, oropharynx, larynx, trachea, and lower airways. Flexible bronchoscopes are also used by anesthetists in endotracheal intubation.

Reusable bronchoscopes may become heavily contaminated with blood and patient secretions during the use and the transmission of pathogenic microorganisms via inadequately decontaminated reusable bronchoscopes has been associated with an increased risk of infection [1].

There are several reports demonstrating the contamination of bronchoscopes after reprocessing; however, it is difficult to estimate what the real risk of infection is because few prospective studies have shown the onset of infection following the use of contaminated bronchoscopes [2,3,4,5,6].

However, during the last few years, several episodes of bronchoscopes-associated outbreaks or pseudo-outbreaks have been reported. Bacteremia and fever following bronchoscopy were reported as transient and not associated with septic complications, but pneumonia after bronchoscopy procedures has been documented with a frequency from 0.02% to 6.3% [6,7].

From January 2010 to June 2015, the US Food and Drug Administration (FDA) received 109 Medical Device Reports (MDRs) about bronchoscope-related infection and device contaminations [8]. Since 2015, the number of MDRs has increased to 100–200 per year and, between 2015 and 2021, the FDA received 867 MDRs associated with bronchoscopes. Among the reports that included the name of specific microorganisms, the most frequently reported organisms were *Mycobacterium*, *Pseudomonas*, *Serratia*, and *Klebsiella*. Of the 867 reports, 7 were reports of deaths. As underlined by FDA, it was unknown whether the reported infections contributed to the patient deaths and whether patient comorbidities may have been a factor [9].

In April 2022, FDA reported five events of cross-contamination in patients undergoing bronchoscopy infected with *Stenotrophomonas maltophilia* and *Pseudomonas aeruginosa* and the same pathogens were found in samples collected from the used bronchoscopes. The incoming inspection of the device detected a perforated bending rubber and a crushed/buckled insertion flexible tube. Additional information received from the hospital, prior to taking remedial action, indicated that the reprocessing procedures did not comply with national regulations and with Pentax instructions for use (IFU) [10].

Although non-adherence to reprocessing guidelines is common, outbreaks associated with bronchoscopy occurred even when reprocessing was carried out correctly [2].

In 2016, Dickson et al. reported an outbreak of gentamicin-resistant *Enterobacter cloacae* and demonstrated the transmission of infection to patients who underwent a bronchoscopy with an endobronchial ultrasound (EBUS) that harbored the pathogen despite reprocessing in accordance with guidelines. In another study, researchers reported that 60% of endoscopes (including bronchoscopes) that were reprocessed in accordance with guidelines had microbial growth, including two Gram-negative species, *S. maltophilia* and *Delftia acidovorans*, from four of tested bronchoscopes [11,12].

Among the reports about specific microorganisms, the most frequently reported were carbapenem-resistant Enterobacteriacae (CRE), such as *Klebsiella pneumoniae*, *Escherichia coli*, and *Enterobacter cloacae*, and related Multi-Drug Resistant Organisms (MDROs), such as particular strains of *Pseudomonas aeruginosa* [8,13].

The estimated average microbial load on bronchoscopes before cleaning was 6.4 × 10^4^ CFU/mL [14]. These devices are more difficult to decontaminate than other endoscopes, as their operating channels are narrower, especially the fibrobronchoscope (1.15–3 mm). The reason for these contaminations is found, in most cases, in inadequate compliance with the manufacturer’s instructions for use (IFUs) and of the bronchoscope-reprocessing guidelines. The FDA reported that some devices were used despite integrity issues (e.g., wall scratches, holes, microlesions) and that in a small percentage of cases, the bronchoscopes remained persistently contaminated, even after a strict observance of the adequate procedure of reprocessing [10]. This critical issue has been pointed out by Ofstead et al., reporting microbial growth in 58% of fully reprocessed bronchoscopes (14/24), including mold, *Stenotrophomonas maltophilia,* and *Escherichia coli/Shigella* species, and visible irregularities were observed in 100% of bronchoscopes [2].

In this context, the microbiological surveillance of reprocessed instruments can be a critical quality control measure to directly assess the efficacy of the reprocessing protocol and to identify those instruments with any lack of integrity in the wall channels that could promote the formation of a biofilm, as well as to allow the indirect verification of the adherence to reprocessing protocols. Specific organisms are used as indicators of weaknesses or mistakes in reprocessing procedures and their isolation is representative of the failed reprocessing phase (e.g., *P. aeruginosa* is indicator of the contamination of the water used for reprocessing or of insufficient drying before endoscope storage).

Recent guidelines recommend a monthly frequency of testing on bronchoscopes. The presence of any quantity of those known as high concern microorganisms (HCO), as *Staphylococcus aureus*, viridians streptococci, non-tuberculous Mycobacteria, enteric or non-enteric Gram-negative or *Candida* species, demonstrate breaches in cleaning and disinfecting procedures; among them, any growth of *Mycobacterium tuberculosis*, *P. aeruginosa*, *Burkholderia cepacia*, or *Acinetobacter baumannii* shows a major breach in this procedure. In case of positivity for HCO, the bronchoscope should be removed from use, the cleaning and disinfection process should be investigated and the device should be tested again; if still positive, structural faults in scope must be considered [15].

Our study aimed to compare the effectiveness of endoscope reprocessing in a Bronchoscopy Unit before and after the introduction of an improved operational procedure that includes regular microbiological surveillance.

## 2. Methods

A cross-sectional study was conducted in the Bronchoscopy Unit (BU) of a large Teaching Hospital located in Pisa with 1082 beds with around 3000 endoscopic procedures and 1100 biopsies/resections per year.

Our study aimed to compare the efficacy of endoscope reprocessing in a Bronchoscopy Unit, between two periods: in 2019, a microbiological surveillance was performed on bronchoscopes to evaluate the decontamination level and analyze the factors associated with non-compliance. Consequently, in 2020, the quality of the process was improved and after the introduction of a new operational procedure, a regular microbiological surveillance was implemented in 2021 in order to evaluate the effectiveness of the reprocessing.

In September 2019, the BU was subject to observational and clinical audits aimed at assessing the quality of the reprocessing and identifying potential critical issues. From 19 November to 11 December, a microbiological sampling on the 14 supplied bronchoscopes and the representative samples of storage environments was performed. After the introduction of an improved procedure in April 2020, in 2021, from 2 March to 22 December, a microbiological surveillance plan was performed quarterly on all the 22 supplied bronchoscopes in order to sample each device at least annually, as already recommended by the manufacturer’s instructions for use, or after the use on patients known to be colonized or infected with multidrug-resistant organisms (MDROs). The surveillance plan included a quarterly sampling of automatic washer-disinfectors and biannually of storage cabinets [16,17].

Additionally, in collaboration with the hospital Clinical Engineer Unit, the age of the use of the bronchoscopes was investigated as a possible factor influencing the contamination level. Due to the reduced surveillance activity during the period of the COVID-19 pandemic, the data obtained in 2020,were not taken into consideration as they were not representative.

### 2.1. Reprocessing Process

In September 2019, the hospital hygiene team observed and audited work practices and procedures involving bronchoscope reprocessing, using the checklist proposed by the Italian guidelines of the Tuscany region [18] (Table 1). Various critical issues were identified; no internal procedure was defined, and the staff carried out reprocessing based on the experience gained and passed on by colleagues. The steps were roughly the same as recommended by the national guidelines: pre-cleaning, leak test, manual cleaning, disinfection, and drying before storage. Bronchoscopes were reprocessed in an automatic washer-disinfector (Soluscope series 4, Soluscope, Aubagne, France), complying with ISO 15883–1/4 and validated annually by an outsourced service of a clinical engineer. However, the timing and the technique used in some of these steps were not optimal. Pre-cleaning was not performed, the timing of manual cleaning was not always compliant with the guidelines because of nurses’ unavailability in the afternoon or during the night (in these cases the cleaning was performed the next morning). Another critical issue was the use of a reusable brush during the manual cleaning, instead of the disposable ones. One of the most critical steps was represented by storage. After a high level disinfection, the external surface of the bronchoscope and the internal channels were blown, for a few seconds, with forced air with an air pistol and directly stored in unventilated cabinet, but most of the bronchoscopes were stored in the transport case; the case was made of durable high impact ABS and lined with polyurethane Pick-N-Pluck™ foam. The entire process was not traceable, neither the high disinfection or the storage timing, nor the patient on whom the specific bronchoscope was used. The lack of traceability created a significant concern regarding the bronchoscope used on patients known colonized or infected by MDROs.

After the audits, several remedial actions were put in place, compliant with the manufacturer’s recommendations and the most recent guidelines, and an improved operational procedure entered into force in April 2020 [19].

Medical and nursing staff assigned to endoscopy were trained to carry out the pre-cleaning procedures correctly. The staff assigned a dedicated healthcare worker trained in reprocessing practice to comply with the timing and modality of all processes, in particular in the case of after-hours and emergency endoscopy.

Single-use brushes, in accordance with manufacture’s instruction, were adopted based on the ESGE/ESGENA recommendation. In order to increase storage hygienic quality, avoiding the use of transport cases, two storage cabinets (DSC800 Soluscope, Aubagne, France) complying with EN ISO 16442:2015 were installed, increasing the bronchoscope shelf life after reprocessing from 72 h to 7 days. To ensure traceability, the report issued by the washer–disinfector was registered to the digital traceability program and a copy of the report was inserted into the patient’s electronic medical record.

### 2.2. Sampling Method and Microbiological Analysis

Microbiological sampling was carried out according to the CDC guidelines in both study periods [20].

The air/water and biopsy channel were each flushed with 10 mL of phosphate Buffered Saline solution 0.01 M (PBS, Merck KGaA, Darmastadt, Germany) with 0.02% of Tween 80 (Merck KGaA, Darmastadt, Germany), then brushed (one brush for each channel), and flushed again with 20 mL of solution for each channel for a total of 60 mL of sample. We used this eluent instead of sterile deionized water because it was considered more suitable for the recovery of viable microorganisms [21].

The final volume of sample (60 mL) was collected in a sterile container added to a same volume of Dey Engley neutralizing broth (DE broth, Sigma Aldrich, Milan, Italy) to neutralize any traces of chemicals which may limit the detection of microorganisms. The entire volume of the sample was filtered through a 0.45 μm filter, the membrane was placed on a plate of Blood Agar (VWR International PBI, Radnor, PA, USA) and incubated at 35–37 °C for 72 h. According to CDC guidelines, colony-forming units (CFU) were enumerated, each species was identified by matrix-assisted laser desorption/ionization time-of-flight mass spectrometry (MALDI-TOF, Bruker Daltonics, Bremen, Germany) to distinguish high-concern organisms (bacteria closely associated with infectious outbreaks) from low/moderate-concern (bacteria not closely associated with infectious outbreaks) organisms [21,22]. In addition, antimicrobial susceptibility tests were performed by Xpert^®^ Carba-R, (Cepheid, Sunnyvale, CA, USA) a real time PCR assay for the rapid detection and differentiation of five genes (blaKPC, blaVIM, blaOXA-48, blaIMP-1, blaNDM) associated with non-susceptibility to carbapenem in Gram-negative bacteria.

The microbiological sampling of washer–disinfectors and the storage cabinet was carried out as previously reported [23].

### 2.3. Non-Compliant Bronchoscopes Assessment

The isolated microorganisms, according to international guidelines and the Italian Position Paper, were distinguished as high-concern (HCO) and low-concern organisms (LCO) [20,22,24]. While HCO are most often associated with diseases and any growth indicate a potential risk of cross-infection (*Mycobacterium tuberculosis*, *P. aeruginosa*, *Burkholderia cepacia* or *Acinetobacter baumannii*), the LCO are less often associated with disease and may result from contamination during sampling or storage (e.g., coagulase-negative staphylococci, micrococci, *Bacillus* spp., diphtheroids). The bronchoscopes are considered non-compliant for any microbial load of HCO (>1 CFU/channels) and higher than 100 CFU/channels of LCO.

**Figure 1:**
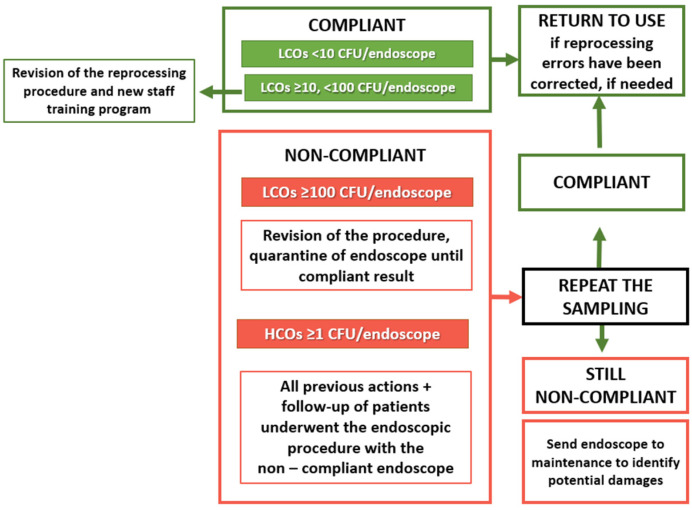
Flowchart of fundamental steps of microbiological surveillance on bronchoscopes to obtain a microbiologically guaranteed and safe device for the patient.

In case of non-compliance, the procedure requires the verification of the reprocessing correct application and a subsequent second sampling. All non-compliant instruments were put in quarantine until a negative result was achieved. In case of persistent non-compliant results, the instrument was sent to the manufacturer’s company, in order to assess the possible presence of micro-lesions of the internal channels. When the number of isolated LCO are >10 and <100 CFU/channels, all mentioned actions are required, except the removal from clinical use, while an LCO load of < 10 CFU/channels does not require any corrective measures (Figure 1).

### 2.4. Statistical Analysis

Differences between storage condition were tested with a non-parametric approach, using Kruskal–Wallis test by ranks, after a logarithmic transformation in base 10 of CFU observed in each group. The multiple comparisons between groups were conducted with Dunn–Šidák correction. A *p* value < 0.05 was considered statistically significant and analyses were performed with GraphPad Prism 5.0 (GraphPad Software, Inc., San Diego, CA, USA). A polynomial function was plotted to observe the relationship between the age of bronchoscopes and the mean microbial load observed. Polynomial regression allows us to highlight better the trend variation in each age point of bronchoscopes analyzed. This method makes a “best fit” line through all the available data points, minimizing the error sum of squares [24]. The contamination rates with their 95% confidence intervals were calculated, and the comparison between the two periods was performed by using the Chi-square test.

## 3. Results

In the first period analyzed in this study (from September to December 2019), 14 of the 22 bronchoscopes of the BU were sampled at least once to test the quality of reprocessing procedures. Out of a total of 14 bronchoscopes, 13 (59.1%; 95% CI = 38.6–79.6) resulted as non-compliant for HCO, 8/13 (36.4%) with a high microbial count (≥100 CFU/all channels). The most frequent microorganism detected was *Staphylococcus aureus*, isolated in 5/13 bronchoscopes (38.5%). *Proteus vulgaris* was isolated in two bronchoscopes (15.4%) and *Enterobacter gergoviae* as well as *Pseudomonas aeruginosa* in one (7.7%). NDM-producing *Klebsiella pneumoniae* was found in two samples collected on the same bronchoscope (15.4%). This bronchoscope was first tested for contamination after use on a known colonized patient, then quarantined and was still found positive for the same microorganism in the second sampling; the endoscope was sent to the manufacturer due to the persistent contamination of internal channels (Table 2). This procedure was applied each time the sampled bronchoscope resulted twice as contaminated by HCO or LCO with a microbial load >100 CFU/mL.

Among the thirteen non-compliant bronchoscopes samples, nine (69%) were collected from bronchoscopes that did not respect the storage recommendations because they were held in a transport case. On the other hand, all samples found to be compliant for microbial contamination were collected from bronchoscopes kept in conditions respecting the storage criteria. Three bronchoscopes (Olympus BF TE, Pentax EB 1530 T3, Pentax FB 18 V) whose samples were confirmed as non-compliant twice consecutively for HCO and high microbial loads (>100 UFC/channels) were sent to the manufacturers for assistance (Table 2).

A significant difference was observed between the microbial load and the storage procedures of the bronchoscopes (Kruskal–Wallis test, *p* = 0.01). The median values observed for the transport case was 2.48 [0.39–2.49] Log (CFU), in a non-ventilated cabinet was 2.48 [0.96–2.48] Log (CFU) and 0.30 [0.08–0.70] Log (CFU) in a ventilated cabinet. The multiple comparison tests were statistically significant between transport cases versus ventilated cabinets (ADJ *p* = 0.03) and non-ventilated cabinets versus ventilated cabinets (ADJ *p* = 0.04).

Regarding the years of use, the correlation observed was low (R = 0.32); the high contamination level detected on two relatively new bronchoscopes (aged 2 and 3 years) decreased the correlation, probably due to the incorrect execution of all reprocessing phases, in particular the pre-cleaning stage that was omitted during the first period of the observation.

**Figure 2:**
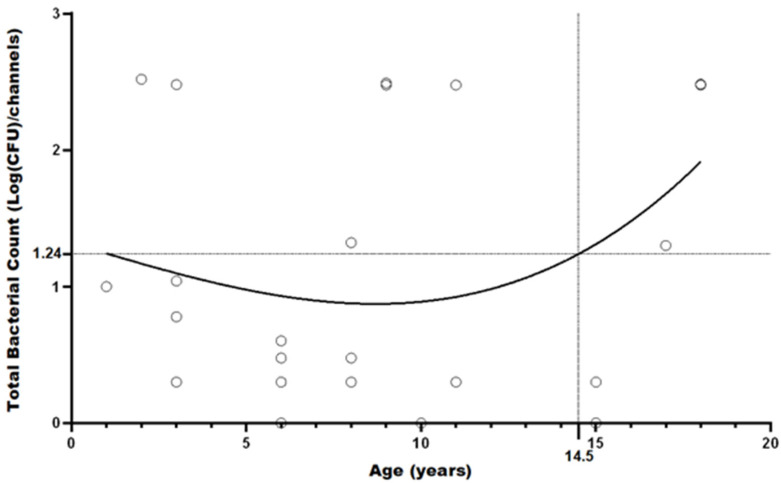
Relationship between the age of bronchoscopes and total bacterial count detected on bronchoscopes.

The level of contamination increases weakly in bronchoscopes that have been in use for more than 14 years (Figure 2).

The inlet and final rinse water of washer–disinfectors and the storage cabinet surfaces were randomly sampled to gain an initial picture of their hygienic status. The water samples collected in one of the two washer–disinfectors was shown to be compliant with the parameters required by the Italian law D.Lgs. 31/2001 and by ISO 15883–4, while the others resulted in non-compliance due to the isolation of *P. aeruginosa* at the inlet of the washer–disinfector.

The analysis conducted on two storage cases detected the presence of *Enterobacter gergoviae* (3 CFU/25 cm^2^) and *Vibrio alginolyticus* (6 CFU/25 cm^2^), respectively. The same HCOs (*E. gergoviae* and *V. alginolyticus)* were isolated from the bronchoscope stored in those storage cases.

Starting in 2021, an implemented microbiological surveillance was regularly performed and from February to November 2021, 31 bronchoscopes, two washer–disinfectors, two storage cabinets complying with EN 16442:2015, and three unventilated vertical cabinet were sampled.

To improve the water quality, and therefore the effectiveness of the microfiltration operated by the automatic washer-disinfector, a pre-filtration system was installed at the entrance of each machine, consisting of three polypropylene filters arranged in series, with porosities of 20, 5, and 1 μm, respectively (Serie Claris, Pall Corporation, PALL, Washington, NY, USA).

After this corrective action, both the quality of the inlet and the rinse water, used by washer–disinfectors, was found to be compliant with standards (eight out of eight samples) as was the hygienic quality of each storage cabinet surfaces (20/20 samples).

Among the 99 samples collected from the 31 bronchoscopes, 13 (13.1%; 95% CI = 6.5–19.8) were non-compliant. These samples showed HCO growth, mainly enterobacteria, including *Klebsiella pneumoniae* as well as Pseudomonadaceae. *K. pneumoniae* was isolated from four out of thirteen samples (30.7%), *P. aeruginosa* and *P. penneri* in two (15.4%), and *E. gergoviae* in one (7.7%) as *A. lwoffii*, *Serratia* spp., *S. aureus*, and *V. alginolyticus.* Two of those bronchoscopes (Olympus-1T180 and Pentax-19 TV) were confirmed as positive twice consecutively for HCO and were sent to the manufacturers for assistance. No bronchoscope was tested positive for *M. tuberculosis* or nontuberculous mycobacteria, despite the previous use on a patient known to be infected with these germs. By comparing the rate of contaminated bronchoscopes following reprocessing between the first and second period, a statistically significant reduction emerged (59.1% versus 13%; *p* < 0.0001)

## 4. Discussion

Bronchoscopy is a common and useful procedure, performed often in patients admitted to intensive care or cardio-thoracic vascular department, often immunocompromised or with comorbidities.

Due to the bronchoscope’s inherent complexity, with its narrower channels in comparison to other types of endoscopes, it represents a challenge in terms of reprocessing. Oropharyngeal pathogens from the upper respiratory tract can remain on the instrument during bronchoscopy and be carried to the following patient.

For this reason, it is essential to perform a regular microbiological surveillance through which the presence of breaches in the manual cleaning and/or in the disinfection process, as well as in handling of bronchoscope, can be verified and so corrected through focused training. This is recommended in several guidelines, included the Australian Guidelines and is well underlined in the Italian multisocieties position paper [22,25].

Although clinical audits contribute to the implementation of the reprocessing procedure, the microbiological analysis was fundamental in our study to highlight the main critical issues and the causes of reprocessing failure. The microbiological analysis permits the identification of microbial indicators, whose presence is the key in determining the not-appropriately-executed reprocessing phase, suggesting the remedial action to apply.

One of the indicators of inadequate reprocessing is represented by *Staphylococcus aureus*, whose isolation is suggestive for the contamination of the hands of operators or of endoscope storage cabinets. *Staphylococcus aureus* was reported in a percentage of 22.7% (5/22) in post-reprocessed bronchoscopes analyzed in the first phase of this investigation. This result is higher than those found in a survey conducted in an Italian Endoscopy Center where a microbiological surveillance was implemented and the post-reprocessing *Staphylococcus* spp. contamination rate was 15.69% [26].

Considering that a different sampling method and analysis was adopted in the cited study, the results obtained in the second phase of our investigation significantly decreased the *S. aureus* isolation rate (1%, 1/99). This result was achieved thanks to the introduction of regular hand hygiene audits, performed through the use of a hand-hygiene digital scan, but also by the improvement of the reprocessing procedure, the introduction of new staff members and periodic staff training. For the success of reprocessing, the presence of a sufficient number of dedicated and competent staff members, who regularly participate in specific and periodically updated training programs, is crucial [27,28].

One of the major critical issues was identified as inadequate storage conditions and no respect of the maximum storage period: many non-conformities were found in bronchoscopes that were stored in transport cases, while all conformities comprised devices kept in conditions that respected the storage criteria. From this aspect emerges the importance of the storage modality in preventing bronchoscopes contamination. The FDA and CDC have recommended a list of supplemental reprocessing measures to further reduce the risk of infection and increase the safety of these medical devices. The guidelines recommend the storage of reprocessed endoscopes in an appropriate manner that prevents recontamination, equipment damages, and the persistence of moisture: the reprocessed endoscopes must be stored in a cabinet of sufficient length, width, and height to allow endoscopes to hang vertically or in a cabinet conforming to the EN 16442 designed by the manufacturer for their horizontal storage [9,20]. 

Although the same number of non-compliant samples were found in the second phase of the study, the sample size and time of surveillance were significantly increased, highlighting the improvement in reprocessing practices after the introduction of the new procedure.

The 2018 ECRI Report underlined how the accumulation of humidity in the internal channels due to the incorrect drying of the instrument, after reprocessing, favors the proliferation of any microbes not eradicated by reprocessing [29].

As prior research on this topic shown, the results of our study may be correlated to residual moisture resulting from the lack of adequate drying and the inappropriate storage of endoscopes that can lead to bacterial proliferation and biofilm formation [28]. A recent study found the persistence of a *P. aeruginosa* strain in a flexible bronchoscope for nearly a year [30]. In our study, we did not find a persistence of contamination, because all bronchoscope tested as non-compliant twice consecutively were sent to manufacturers for assistance.

However, even with complete compliance with the reprocessing guidelines and the manufacturers’ instructions for use, a variable percentage of bronchoscopes can remain contaminated after reprocessing with a high level of disinfection [31]. In our study, frequent and regular microbiological surveillance permitted the identification of contaminated bronchoscopes, despite the execution of the correct reprocessing procedure, thus allowing their clinical use to be halted and reducing the rate of non-compliant instruments from 59 to 12%. The rationale in performing a regular microbiological surveillance is highlighted by different guidelines, which recommend it with different periodicity. A quarterly frequency for the analyses of endoscopes and washer–disinfectors is proposed by Dutch [32] and German [33] guidelines, while the Australian [15] studies recommend a yearly frequency and the Italian position paper every 3–6 months [25]. Moreover, there is no uniformity among the guidelines regarding the quarantine of endoscope until the availability of negative microbiological results.

The level of contamination found on the bronchoscopes was weakly correlated with the years of use only in the older bronchoscopes used for over 14 years. Microlesions, due to the passage of accessories in the operative channel, can be the cause of biofilm formation and therefore of the persistent contamination of bronchoscopes. Only through the visual analysis of the channels with a borescope is it possible to confirm the presence of the microlesions. Our data were limited and did not allow us to find a highly significant correlation.

In recent years, some studies have evaluated the possibility of introducing disposable bronchoscopes, evaluating their performance, safety, and cost-effectiveness. In care settings where an insufficient rate of adequately reprocessed endoscopes is detected and the risk of cross-contamination may be high, the use of single-use bronchoscopes may be an adequate solution in selected frail patients, after the cost–benefit analysis and the epidemiological assessment of MDRO colonization/infection within the care setting. This recommendation was stated by the FDA 2021 safety communications that also suggested the use of the disposable bronchoscope when there is no support for immediate reprocessing [9]. Moreover, their use was recommended, when available, during the COVID-19 pandemic, due to the importance of reducing the occupational risk associated with each aerosol-generating practices, such as the operator-dependent steps of reprocessing [34].

The use of disposable bronchoscopes is not the only strategy to reduce the risk for operators and environmental contamination. A combination of engineering solutions, such as automated washer–disinfectors or physical barriers, including personal protective equipment (PPE) and splash/exposure, would be necessary to reduce personnel aerosol exposure in endoscopy units. New data published in a recent study, in fact, suggest that personnel who process reusable endoscopes may be frequently exposed to tissue, blood, and patient fluids even when wearing recommended PPE. The first real-setting evaluation of PPE effectiveness for reprocessing personnel found that PPE does not provide adequate protection [35].

Our study is one of the few in which the effectiveness of bronchoscope reprocessing was evaluated. As of 2018, the FDA has issued an alert regarding bronchoscope-related transmission risks, which was previously considered to be lesser than the risk linked to the use of duodenoscopes.

Although our study presents some limitations, as the scope of the data is constrained to a single BU and the microbiological surveillance restricted to one year of observation, our preliminary results support the usefulness of microbiological surveillance in reducing the contamination of bronchoscopes and hence the potential associated infection risk.

The data did not include the patient notification and follow-up as requested by guidelines because the application procedures to the ethic committee have not yet been started; a follow-up study is planned, focused on this topic in particular.

The correlation between age and the microbial contamination of bronchoscopes, already demonstrated in the literature, was not shown as statistically significant in our study.

It might be interesting to statistically compare the HCO detection frequency and microbial concentration between the two periods. However, a time series analysis would require a higher number of observations. In our case, the sample size of the first period is not adequate to perform such research, and for this reason we carried out a descriptive analysis.

## 5. Conclusions

Our data showed a high percentage of contaminated devices before the adoption of remedial actions. Once the process was audited and the recommendations were strictly adopted, the improved results obtained in terms of conformity rate demonstrated that the application of all correct steps of reprocessing, including the correct storage, plays a key role in reducing the instrument’s contamination and the potential risk of cross-contamination.

## Figures and Tables

**Table 1:** Checklist of the reprocessing process and critical issue observed before the remedial actions.

Phase	Performed	Not Performed
Pre-cleaning is performed immediately after the procedure		X
The endoscope is cleaned externally using sponge or lint-free cloth soaked in detergent solution	X	
The endoscope is cleaned internally with the detergent solution aspirated through all the channels until it is macroscopically cleaned.	X	
The dirty endoscope is transported to the reprocessing room in the recommended time		X
The dirty endoscope is transported using a container that is completely closed, resistant to punctures, and labeled to indicate biohazard	X	
Before cleaning, the leak test is performed	X	
The detergent solution indicated by the manufacturer is used and the contact times is respected	X	
Appropriately sized brushes are used as indicated by the manufacturer	X	
The brushes are passed in all channels, valves, and all removable parts of the instrument	X	
Brushes are disposable (or if reusable they are disinfected/sterilized after each use)		X
Endoscope and all removable parts are rinsed and dried	X	
The high-level disinfection of the endoscopes is performed	X	
After high level disinfection, the internal channels are dried with compressed air for medical use	X	
The clean endoscope is transported to the storage room in a container completely closed, resistant to punctures	X	
The endoscope with valves removed are stored in a dedicated cabinet		X
Each reprocessing steps is traced		X

**Table 2:** Bronchoscope model, age, storage condition, and high-concern organisms identified during the microbiological surveillance conducted in the first and second phase of the study, respectively; November–December 2019 and February–November 2021.

First Phase						
Bronchoscope Model	Age (Years)	Storage Condition	Sampling Date	Time after Disinfection (h)	Microorganism	Total Bacterial Count (CFU/Channels)
**OLYMPUS BF TE** **(Routine testing)**	9	Transport case	19 November	120	*NDM-K.pneumoniae*, *Staphylococcus* spp.	310
**OLYMPUS BF TE** **(Retesting for non-conformity)**	9	Non-ventilated Cabinet	27 November	1	*NDM-K.pneumoniae*, *Staphylococcus* spp.	300
**PENTAX EB 1530 T3** **(Routine testing)**	18	Transport case	27 November	140	*E. gergoviae*, *Staphylococcus* spp.	300
**PENTAX FB 18 BS** **(Routine testing)**	6	Transport case	27 November	-	*Proteus vulgaris*	1
**PENTAX FB15 BS** **(Routine testing)**	2	Transport case	27 November	120	*S. aureus*	331
**FUJIFILM EB-530S** **(Routine testing)**	3	Transport case	27 November	1	*Enterobacter gergoviae*, *Staphylococcus* spp.	11
**PENTAX EB 1530 T3** **(Retesting for non-conformity)**	18	Transport case	5 December	168	*S. multivorum*	304
**PENTAX FB 15 BS** **(Routine testing)**	3	Transport case	5 December	144	*S. aureus*	302
**PENTAX FB 18 BS** **(Routine testing)**	6	Transport case	5 December	120	*S. aureus*	3
**PENTAX FB 18 V** **(Retesting for non-conformity)**	6	Transport case	5 December	48	*S. aureus*	2
**OLYMPUS BF 1T 180** **(Routine testing)**	11	Non-ventilated Cabinet	5 December	21	*P. aeruginosa*, *Providencia stuartii*, *S. maltophilia*, *Vibrio alginolyticus*	300
**PENTAX FB 18 V** **(Routine testing)**	6	Non-ventilated Cabinet	11 December	24	*Proteus vulgaris*	4
**OLYMPUS BF 1T 180** **(Routine testing)**	11	Non-ventilated Cabinet	11 December	43	*S. aureus*, *Proteus penneri*, *S. paucimobilis*	300
**SECOND PHASE**						
**PENTAX FB 18 BS** **(Routine testing)**	3	Ventilated Cabinet	2 March	1	*Serratia* spp.	6
**OLYMPUS BF-1T180** **(Retesting for non-conformity)**	15	Ventilated Cabinet	24 May	48	*K. pneumoniae*	1
**PENTAX 19 TV** **(Retesting for non-conformity)**	10	Ventilated Cabinet	24 May	72	*K. pneumonae*	1
**PENTAX 19 TV** **(Retesting for non-conformity)**	10	Ventilated Cabinet	28 May	96	*A. lwoffii*	1
**OLYMPUS BF 1T180** **(Retesting for non-conformity)**	15	Ventilated Cabinet	28 May	3	*P. aeruginosa*	2
**PENTAX 18 V** **(Routine testing)**	11	Ventilated Cabinet	30 June	24	*K.pneumoniae*	2
**PENTAX FB 15 BS** **(Routine testing)**	8	Non-ventilated Cabinet	30 June	24	*P. aeruginosa*	21
**FUJIFILM EB-530S** **(Routine testing)**	3	Ventilated Cabinet	30 August	48	*K. pneumoniae*	2
**PENTAX 18 P** **(Routine testing)**	17	Ventilated Cabinet	28 September	24	*S.aureus*	20
**FUJIFILM EB-530T** **(Routine testing)**	3	Ventilated Cabinet	5 October	96	*V. alginolyticus*	2
**STORZ 11301BNI** **(Routine testing)**	8	Ventilated Cabinet	19 October	4	*P. penneri*	3
**FUJIFILM EB-530 US** **(Routine testing)**	1	Ventilated Cabinet	9 November	4	*P. penneri*	10
**PENTAX FB 15 BS** **(Routine testing)**	8	Ventilated Cabinet	9 November	4	*E. gergoviae*	2

## Data Availability

The data were collected and processed directly by the authors, so we have no link to suggest.

## References

[1] Manthous C., Tobin M. (2015). Flexible bronchoscopy. Am. J. Respir. Crit. Care Med..

[2] Ofstead C.L., Quick M.R., Wetzler H.P., Eiland J.E., Heymann O.L., Sonetti D.A., Ferguson J.S. (2018). Effectiveness of Reprocessing for Flexible Bronchoscopes and Endobronchial Ultrasound Bronchoscopes. Chest.

[3] Zweigner J., Gastmeier P., Kola A., Klefisch F.R., Schweizer C., Hummel M. (2014). A carbapenem resistant *Klebsiella pneumoniae* outbreak following bronchoscopy. Am. J. Infect. Control.

[4] DiazGranados C.A., Jones M.Y., Kongphet-Tran T., White N., Shapiro M., Wang Y.F., Ray S.M., Blumberg H.M. (2009). Outbreak of *Pseudomonas aeruginosa* infection associated with contamination of a flexible bronchoscope. Infect. Control. Hosp. Epidemiol..

[5] Kovaleva J., Peters F.T., van der Mei H.C., Degener J.E. (2013). Transmission of infection by flexible gastrointestinal endoscopy and bronchoscopy. Clin. Microbiol. Rev..

[6] Mehta A.C., Muscarella L.F. (2020). Bronchoscope-Related “Superbug” Infections. Chest.

[7] Sato Y., Murata K., Yamamoto M., Ishiwata T., Kitazono-Saitoh M., Wada A., Takamori M. (2020). Risk factors for post-bronchoscopy pneumonia: A case-control study. Sci. Rep..

[8] FDA (2015). Safety Communications & Infections Associated with Reprocessed Flexible Bronchoscopes: FDA Safety Communication Infections Associated with Reprocessed Flexible Bronchoscopes: FDA Safety Communication. http://www.fda.gov/MedicalDevices/Safety/AlertsandNotices/ucm462949.htm.

[9] (2021). Flexible Bronchoscopes and Updated Recommendations for Reprocessing: FDA Safety Communication FDA. https://www.fda.gov/medical-devices/safety-communications/flexible-bronchoscopes-and-updated-recommendations-reprocessing-fda-safety-communication.

[10] PENTAX (2017). MAUDE Adverse Event Report: Hoya Corporation PENTAX Tokyo Office PENTAX Fiber Bronchoscope.

[11] Dickson A. Enterobacter cloacae found in an endobronchial ultrasound scope (EBUS): Now what do we do?. Proceedings of the Association for Professionals in Infection Control and Epidemiology Annual Conference.

[12] Portland O.R., Ofstead C.L., Doyle E.M., Eiland J.E., Amelang M.R., Wetzler H.P., England D.M., Mascotti K.M., Shaw M.J. (2016). Practical toolkit for monitoring endoscope reprocessing effectiveness: Identification of viable bacteria on gastroscopes, colonoscopes, and bronchoscopes. Am. J. Infect. Control.

[13] Galdys A.L., Marsh J.W., Delgado E., Pasculle A.W., Pacey M., Ayres A.M., Metzger A., Harrison L.H., Muto C.A. (2019). Bronchoscope-associated clusters of multidrug-resistant *Pseudomonas aeruginosa* and carbapenem-resistant *Klebsiella pneumoniae*. Infect. Control Hosp. Epidemiol..

[14] Zhang Y., Zhou H., Jiang Q., Wang Q., Li S., Huang Y. (2020). Bronchoscope-related *Pseudomonas aeruginosa* pseudo-outbreak attributed to contaminated rinse water. Am. J. Infect. Control.

[15] Cowen A., Jones D., Wardle E. (2010). Infection Control in Endoscopy.

[16] Saliou P., Le Bars H., Payan C., Narbonne V., Cholet F., Jézéquel J., Scotet V., Robaszkiewicz M., Cornec D., Héry-Arnaud G. (2016). Measures to improve microbial quality surveillance of gastrointestinal endoscopes. Endoscopy.

[17] Bisset L., Cossart Y.E., Selby W., West R., Catterson D., O’Hara K., Vickery K. (2006). A prospective study of the efficacy of routine decontamination for gastrointestinal endoscopes and the risk factors for failure. Am. J. Infect. Control.

[18] Agenzia Regionale di Sanità Toscana (ARS) (2013). Il Reprocessing in Endoscopia Digestiva: Criticità e Strumenti per la Sicurezza del Percorso. Collana dei Documenti ARS, n. 70. https://www.ars.toscana.it/files/pubblicazioni/Volumi/2013/doc_ars_70_2013_ok.pdf.

[19] Beilenhoff U., Biering H., Blum R., Brljak J., Cimbro M., Dumonceau J.M., Hassan C., Jung M., Kampf B., Neumann C. (2018). Reprocessing of flexible endoscopes and endoscopic accessories used in gastrointestinal endoscopy: Position Statement of the European Society of Gastrointestinal Endoscopy (ESGE) and European Society of Gastroenterology Nurses and Associates (ESGENA)—Update 2018. Endoscopy.

[20] Centers for Disease Control and Prevention (CDC) Duodenoscope Surveillance Sampling & Culturing (2018). Reducing the Risks of Infection. https://www.fda.gov/media/111081/download.

[21] Centers for Disease Control and Prevention (CDC) (2016). Essential Elements of a Reprocessing Program for Flexible Endoscopes—Recommendations of the Healthcare Infection Control Practices Advisory Committee.

[22] South Australia Health Safety & Quality Strategic Governance (2017). Clinical Guideline for Microbiological Testing of Endoscopes. https://www.sahealth.sa.gov.au.

[23] Casini B., Tuvo B., Marciano E., Del Magro G., Gemignani G., Luchini G., Cristina M., Costa A., Arzilli G., Totaro M. (2021). Improving the Reprocessing Quality of Flexible Thermolabile Endoscopes: How to Learn from Mistakes. Int. J. Environ. Res. Public Health.

[24] Ostertagova E. (2021). Modelling using polynomial regression. Procedia Eng..

[25] Casini B., Pan A., Guarini A., Rivara C., Zullo A., Monica F., Cimbro M., Casarano S., Inglese A., Vaghi A. (2021). Multisocieties position paper: Microbiological surveillance on flexible endoscopes. Dig Liver Dis..

[26] Troiano G., Lo Nostro A., Calonico C., Nante N., Magistri L., Pulci M.B., Niccolini F. (2019). Microbiological surveillance of flexible bronchoscopes after a high-level disinfection with peracetic acid: Preliminary results from an Italian teaching hospital. Ann. Ig..

[27] Perumpail R.B., Marya N.B., McGinty B.L., Muthusamy V.R. (2019). Endoscope reprocessing: Comparison of drying effectiveness and microbial levels with an automated drying and storage cabinet with forced filtered air and a standard storage cabinet. Am. J. Infect. Control.

[28] Ofstead C.L., Heymann O.L., Quick M.R., Eiland J.E., Wetzler H.P. (2018). Residual moisture and waterborne pathogens inside flexible endoscopes: Evidence from a multisite study of endoscope drying effectiveness. Am. J. Infect. Control.

[29] ECRI Institute (2018). Top 10 Health Technology Hazards for 2018: Executive Brief. https://www.ecri.org/Resources/Whitepapers_and_reports/Haz_18.pdf.

[30] Aumeran C., Thibert E., Chapelle F.A., Hennequin C., Lesens O., Traoré O. (2012). Assessment on experimental bacterial biofilms and in clinical practice of the efficacy of sampling solutions for microbiological testing of endoscopes. J. Clin. Microbiol..

[31] Ofstead C.L., Hopkins K.M., Buro B.L., Eiland J.E., Wetzler H.P. (2020). Challenges in achieving effective high-level disinfection in endoscope reprocessing. Am. J. Infect. Control.

[32] Dutch Advisory Board Cleaning and Disinfection Flexible Endoscopes (SFERD) (2017). Flexible Endoscopes. In Professional Standard Handbook Cleaning and Disinfection; Version 4.1. http://www.infectiepreventieopleidingen.nl/downloads/SFERDHandbook3_1.pdf.

[33] (2012). Empfehlung der Kommission fur Krankenhaushygiene und In- fektionspravention (KRINKO) beim Robert-Koch- Institut (RKI) und des Bundesinstituts fur Arzneimittel und Medizin- produkt (BfArM). Anforderungen Die Hyg. Bei Der Auf Bereitung Von Med. Bundesgesundheitsbl.

[34] Wahidi M.M., Lamb C., Murgu S., Musani A., Shojaee S., Sachdeva A., Maldonado F., Mahmood K., Kinsey M., Sethi S. (2020). American Association for Bronchology and Interventional Pulmonology (AABIP) statement on the use of bronchoscopy and respiratory specimen collection in patients with suspected or confirmed COVID-19 infection. J. Bronchol. Interv. Pulmonol..

[35] Ofstead C.L., Hopkins K.M., Smart A.G., Brewer M.K. (2022). Droplet dispersal in decontamination areas of instrument reprocessing suites. Am. J. Infect. Control.

